# Effect of Stenting Strategy on the Outcome in Patients with Non-Left Main Bifurcation Lesions

**DOI:** 10.3390/jcm11195658

**Published:** 2022-09-26

**Authors:** Yongwhan Lim, Min Chul Kim, Youngkeun Ahn, Doo Sun Sim, Young Joon Hong, Ju Han Kim, Myung Ho Jeong, Hyeon-Cheol Gwon, Hyo-Soo Kim, Seung Woon Rha, Jung Han Yoon, Yangsoo Jang, Seung-Jea Tahk, Ki Bae Seung

**Affiliations:** 1 Department of Cardiology, Chonnam National University School of Medicine, Chonnam National University Hospital, Gwangju 61469, Korea; 2 Division of Cardiology, Department of Internal Medicine, Samsung Medical Center, Seoul 06351, Korea; 3 Division of Cardiology, Department of Internal Medicine, Seoul National University Hospital, Seoul 03080, Korea; 4 Division of Cardiology, Department of Internal Medicine, Korea University Guro Hospital, Seoul 08308, Korea; 5 Division of Cardiology, Department of Internal Medicine, Wonju Christian Hospital, Wonju 22070, Korea; 6 Division of Cardiology, Department of Internal Medicine, Yonsei University Severance Hospital, Seoul 03722, Korea; 7 Division of Cardiology, Department of Internal Medicine, Ajou University Hospital, Suwon 16499, Korea; 8 Division of Cardiology, Department of Internal Medicine, Catholic University Kangnam, St. Mary’s Hospital, Seoul 06591, Korea

**Keywords:** Bifurcation, percutaneous coronary intervention, non-left main bifurcation, provisional one-stent, elective two stent

## Abstract

Previous studies have not compared outcomes between different percutaneous coronary intervention (PCI) strategies and lesion locations in non-left main (LM) bifurcation lesions. We enrolled 2044 patients from a multicenter registry with an LAD bifurcation lesion (n = 1551) or non-LAD bifurcation lesion (n = 493). The primary outcome was target lesion failure (TLF), a composite of cardiac death, myocardial infarction, and target lesion revascularization (TLR). During a median follow-up period of 38 months, non-LAD bifurcation lesions treated with the two-stent strategy, compared with the one-stent strategy, were associated with more frequent TLF (20.7% vs. 6.3%, *p* < 0.01), TLR (16.7% vs. 4.7%, *p* < 0.01), and target vessel revascularization (TVR; 18.2% vs. 6.3%, *p* < 0.01). There was no significant difference in outcome among LAD bifurcation lesions treated with different PCI strategies. The two-stent strategy was associated with a higher risk of TLF (adjusted HR 4.34, CI 1.93–9.76, *p* < 0.01), TLR (adjusted HR 4.30, CI 1.64–11.27, *p* < 0.01), and TVR (adjusted HR 5.07, CI 1.69–9.74, *p* < 0.01) in the non-LAD bifurcation lesions. The planned one-stent strategy is preferable to the two-stent strategy for the treatment of non-LAD bifurcation lesions.

## 1. Introduction

Coronary bifurcation lesions are encountered in 30% of percutaneous coronary intervention (PCI) procedures [1]. Even with the use of second-generation drug-eluting stents (DESs), outcomes after PCI are worse for coronary bifurcation lesions compared with non-bifurcation lesions [2]. There is no agreement on the ideal management of coronary bifurcation lesions.

Although there are many stenting strategies for bifurcation lesions [3], they could be classified into the provisional approach or the planned one-stent and elective two-stent strategies.

In the planned one-stent strategy, the main branch (MB) is always stented, but the side branch (SB) is stented only if judged necessary by the operator. In contrast, the operator intends to stent both the MB and SB in the elective two-stent strategy.

Previous studies comparing the planned one-stent and elective two-stent strategies reported mixed results. In comparison with the elective two-stent strategy, different studies have found better [4,5,6], similar [7,8], or worse [9,10,11] outcomes after the planned one-stent strategy. A recent meta-analysis also showed mixed results [12,13,14].

Based on accumulated evidence, current guidelines generally recommend the elective two-stent strategy only in selected cases based on the anatomy of a bifurcation lesion or on the operator’s experience [15]. When choosing an ideal PCI strategy for a bifurcation lesion, some have suggested that the location of the bifurcation lesion might be one factor affecting outcomes after PCI, and some data showed different impacts of stenting strategies according to bifurcation location [16,17,18].

No previous studies, however, have compared outcomes after PCI of non-LM bifurcation lesions, which include either left anterior descending (LAD) or non-LAD coronary arteries according to stenting strategies. We compared the outcomes of PCI for bifurcation lesions affecting the left anterior descending (LAD) and non-LAD coronary arteries treated with stenting with different strategies: planned one-stent or elective two-stent.

## 2. Materials and Methods

### 2.1. Study Population

The Coronary Bifurcation Stenting (COBIS) Registry II is a multicenter, observational registry collecting information on the management and outcomes of bifurcation lesions treated with PCI using DESs. Between January 2003 and December 2009, 2897 patients with coronary bifurcation lesions were enrolled from 18 PCI centers in South Korea. Patients were included in this study if they had a bifurcation lesion, MB diameter ≧ 2.5 mm, and SB diameter ≧ 2.3 mm. Patients with cardiogenic shock, a history of cardiopulmonary resuscitation, or protected LM artery disease were excluded. The baseline characteristics of the patients and findings of the PCI were documented and analyzed at the core laboratory (Cardiac and Vascular Center, Samsung Medical Center, Seoul, Korea). We have previously published the details of our methodology and analysis [18,19]. The ethics committees at each participating center approved the study protocol, and all procedures followed the principles of the Declaration of Helsinki. All patients provided written informed consent prior to participation in the registry.

Of the 2897 patients in the COBIS II registry, 2044 patients (70.6%) with non-LM bifurcation lesions were included in this study. These 2044 patients were divided into two groups based on the lesion location: LAD (n = 1551) group and non-LAD (n = 493) group. Each group was subdivided into two subgroups based on the PCI strategy used: a one-stent strategy subgroup and a two-stent strategy subgroup. The subdivision of patients into one-stent strategy and two-stent strategy subgroups was based on operator intention at the start of the procedure.

### 2.2. Study Definitions and Outcomes

All deaths were considered to be of a cardiac cause unless a definite non-cardiac cause of death could be established. Myocardial infarction (MI) was defined as an increased level of creatine kinase–MB fraction or troponin-T/troponin-I and symptoms or electrocardiography findings indicative of MI. Periprocedural elevation of cardiac enzymes was disregarded. Target lesion revascularization (TLR) was defined as the need for repeat PCI of the lesion within 5 mm of the deployed stent or bypass graft surgery of the target vessel. Definite, probable, and possible stent thrombosis were defined according to Academic Research Consortium recommendations [20]. Target vessel revascularization (TVR) was defined as the need for repeat PCI of the vessel that was stented during the index PCI. Chronic kidney disease was defined as either an estimated glomerular filtration rate (GFR) of ≤60 mL/min/1.73 m^2^ or the need for renal replacement therapy. We have previously published the operational definitions used in the COBIS II registry for angiographic findings [19]. The primary outcome in this study was target lesion failure (TLF), which was the composite of cardiac death, acute MI, and TLR. Secondary outcomes were the individual components of the primary endpoint, TVR, and definite stent thrombosis.

### 2.3. Statistical Analysis

Categorical variables were analyzed using the chi-square or Fisher’s exact test. Continuous variables were expressed as the mean ± SD or median and interquartile range. Continuous variables were analyzed using the Student’s *t*-test or Wilcoxon rank-sum test. *p* values were two-tailed, and *p* < 0.05 was considered statistically significant. Kaplan–Meier curves were used to compare the primary and secondary outcomes between different bifurcation locations and treatment strategies. Differences in survival between comparison groups were assessed using the log-rank test.

The Cox proportional-hazards regression model was used to test whether the elective two-stent strategy is an independent predictor of clinical outcomes. For multivariate analysis, we used variables that appeared significant in the univariate analysis (*p* < 0.1) and variables with a known effect on outcomes, including age, diabetes mellitus, hypertension, dyslipidemia, acute coronary syndrome, chronic kidney disease, previous bypass surgery or PCI, left ventricle ejection fraction ≤ 50%, multi-vessel disease, calcified MB or SB, true bifurcation, use of intravascular ultrasound (IVUS), transradial approach for PCI, and final kissing balloon inflation. These variables were used to calculate the propensity score for being assigned to either PCI strategy. Final kissing balloon inflation was excluded from this calculation because it varies between the PCI strategies. For both the LAD and non-LAD bifurcation groups, propensity score matching with a 2:1 ratio was implemented between the planned one-stent and elective two-stent subgroups. The adjusted hazard ratio (HR) was calculated for both treatment strategies. The standardized mean difference (SMD) was calculated for the adjusted variables (Table S1 and Figure S1); an SMD < 0.1 was considered acceptable based on previous studies [19,21].

All statistical analyses were performed using the R statistical package (version 4.0.3; R Foundation for Statistical Computing, Vienna, Austria; https://www.R-project.org).

## 3. Results

### 3.1. Baseline Characteristics

The baseline characteristics of the patients, lesions, and procedures are described in Table 1.

Of the 2044 patients with non-LM bifurcation lesions included in this study, 426 patients (20.8%) were treated with the two-stent strategy. Compared to LAD bifurcation lesions, non-LAD bifurcation lesions were treated less frequently with the two-stent strategy (23.2% vs. 13.3%, respectively). Patients in the one-stent and two-stent subgroups of the LAD bifurcation group had similar baseline characteristics, except for a higher number of patients with previous PCI in the two-stent subgroup compared with the one-stent subgroup (9.5% vs. 14.4%, respectively, *p* = 0.01). An insignificant increase in the number of patients with previous PCI was also seen in the two-stent subgroup compared with the one-stent subgroup of the non-LAD bifurcation group (13.3% vs. 22.7%, *p* = 0.069).

The proportion of true bifurcation lesions was higher in the elective two-stent groups regardless of lesion location (53.1% vs. 76.1% in LAD bifurcation, *p* < 0.01 and 42.4% vs. 84.8% in non-LAD bifurcation, *p* < 0.01). Mini-Crush and T-stenting were the most frequently used techniques in both groups. IVUS was used more frequently during the two-stent technique than one-stent technique in both groups (29.1% vs. 49.4% and 20.4% vs. 37.9%, respectively). IVUS was used more frequently for LAD bifurcation lesions treated with the two-stent strategy than for non-LAD bifurcation lesions treated with the two-stent strategy (49.4% vs. 37.9%, respectively). Among patients treated with the planned one-stent strategy, SB was stented more frequently in the non-LAD bifurcation group than in the LAD bifurcation group (6.8% vs. 2.9%, respectively). There were no significant differences in SB total stent length and maximal stent diameter between the one-stent and two-stent strategies.

### 3.2. Quantitative Analysis of PCI

The results of our quantitative analysis of PCI are described in Table 2.

The reference diameters of each part of the bifurcation lesions were similar in all groups. The pre-intervention angles between the MB and SB were significantly narrower in the LAD bifurcation group than in the non-LAD bifurcation group (55.0 ± 18.2 vs. 60.9 ± 21.0, respectively; Table S2). Among lesions treated with the two-stent strategy, the angles between the MB and SB in the non-LAD bifurcation group were wider than in the LAD bifurcation group (56.5 ± 20.4 vs. 52.1 ± 16.6, respectively).

In comparison with lesions treated with the one-stent strategy in the LAD and non-LAD bifurcation groups, those treated with the two-stent strategy had more severe SB ostium stenosis (diameter: 43.8 ± 22.3% vs. 58.4 ± 20.3% and 38.0 ± 23.7% vs. 60.7 ± 23.9%, respectively) and a longer SB lesion length (4.4 ± 6.2 mm vs. 10.8 ± 8.5 mm and 2.9 ± 5.1 mm vs. 12.3± 10.7 mm, respectively). Post-intervention residual stenosis at and distal to the SB ostium was less severe in the two-stent strategy subgroup compared with the one-stent strategy subgroup in both the LAD and non-LAD bifurcation groups (43.7 ± 21.5% vs. 8.9 ± 12.5% and 37.0 ± 22.2% vs. 8.3 ± 10.9%, respectively).

### 3.3. Clinical Outcomes

**Figure 1:**
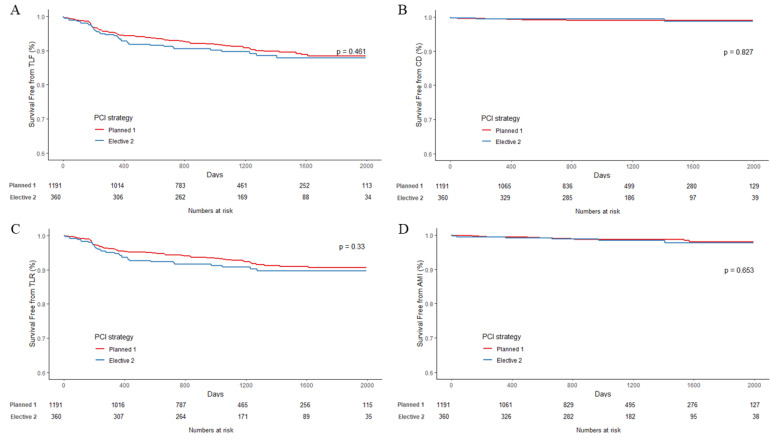
Kaplan–Meier curves in patients with LAD bifurcation lesions according to PCI strategies: planned one-stent (red) or elective two-stent (green). Curves are for (**A**) TLF, (**B**) cardiac death, (**C**) AMI, and (**D**) TLR, respectively. AMI = acute myocardial infarction; PCI = percutaneous coronary intervention; TLF = target lesion failure; TLR = target lesion revascularization.

**Figure 2:**
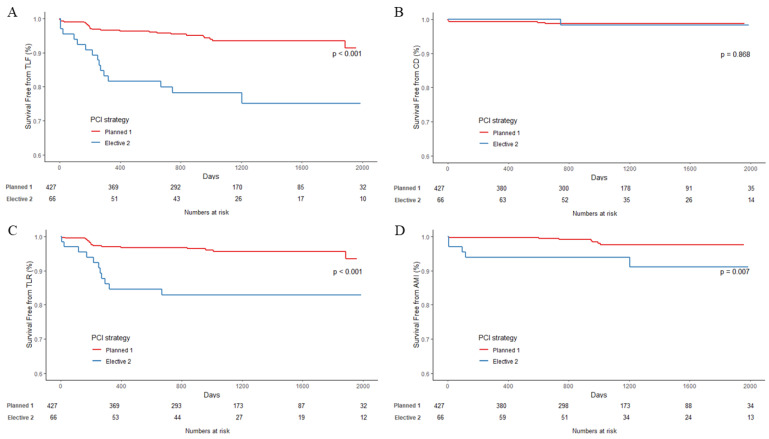
Kaplan–Meier curves in patients with non-LAD bifurcation lesions according to PCI strategies: planned one-stent (red) or elective two-stent (green). Curves are for (**A**) TLF, (**B**) cardiac death, (**C**) AMI, and (**D**) TLR, respectively. AMI = acute myocardial infarction; PCI = percutaneous coronary intervention; TLF = target lesion failure; TLR = target lesion revascularization.

The clinical outcomes are described in Table 3. The median duration of follow-up was 38 months (interquartile range 25–52 months) (36.9 months, interquartile range 25.3–51.9 months for the LAD bifurcation group and 37.3 months, interquartile range 25.1–52.9 months for non-LAD bifurcation group). In the LAD bifurcation group, there were no statistically significant differences in primary and secondary outcomes between the one-stent and two-stent strategy subgroups. In the non-LAD bifurcation group, TLF occurred more frequently in patients treated with the two-stent strategy than those treated with the one-stent strategy (6.3% vs. 22.7%, respectively, *p* < 0.01). The more frequent occurrence of the composite outcome in the non-LAD bifurcation group than in the LAD bifurcation group was attributable to the higher incidence of TLR (1.6% vs. 7.6%, respectively, *p* = 0.013) and TVR (6.3% vs. 18.2%, respectively, *p* < 0.01). Kaplan–Meier curves showed significant differences in TLF, AMI, and TLR in the non-LAD bifurcation group between both PCI strategies (Figure 1 and Figure 2). However, there was no significant difference between the subgroups in the LAD bifurcation lesion group.

The adjusted HR was calculated using the Cox proportional hazards model (Table 2). The two-stent strategy was associated with a higher risk of TLF (adjusted HR 4.34, 95% CI 1.93–9.76, *p* < 0.01), TLR (adjusted HR 4.30, 95% CI 1.64–11.27, *p* < 0.01), and TVR (adjusted HR 5.07, 1.69–9.74, *p* < 0.01) in the non-LAD bifurcation group. These associations remained statistically significant even after propensity score matching (Table 2). The adjusted HRs for outcomes in the LAD group were not statistically significant both before and after propensity score matching.

## 4. Discussion

We compared the outcomes between the planned one-stent and elective two-stent strategies of PCI for LAD and non-LAD bifurcation lesions in the COBIS II registry. There was a higher risk of TLF, TLR, and TVR in the non-LAD bifurcation group treated with the elective two-stent strategy. The outcomes after PCI for LAD bifurcation lesions were similar between the one-stent and two-stent strategies. To the best of our knowledge, this is the first study to compare the outcomes after PCI between LAD and non-LAD bifurcation lesions using different strategies.

Most previous studies included 16% [4] to 24% [6] patients with non-LAD lesions, but did not analyze the results according to the location of the lesion. A similar proportion of patients with non-LAD bifurcation lesions was seen in our registry and reflects the proportion in the real world (24.1%). Some retrospective studies classified the bifurcation location into LM and non-LM, but did not divide further into LAD and non-LAD [16,17,18].

We found a high rate of TLF in non-LAD bifurcation lesions treated with the two-stent strategy in our study. This may have been due to the less frequent use of final kissing balloon inflation in the non-LAD group treated with the two-stent technique compared to the LAD group (78.8% vs. 82.2%, respectively). Previous studies have suggested a beneficial effect of final kissing balloon inflation in various two-stent strategies [9,22,23,24,25]. Due to the significant benefits of final kissing balloon inflation on clinical outcomes, many experts consider its use to be mandatory [26]. However, other studies conducted during the same period as our study also had less frequent use of final kissing balloon inflation [4,6], and the use varied in different subgroups of those studies. Song et al. [18] found more frequent use of final kissing balloon inflation during the two-stent strategy in the LM bifurcation lesion group (89.5%) compared with the non-LM bifurcation lesion group (81.7%) in the COBIS II registry. The less frequent use of final kissing balloon inflation in our study may have been responsible for the high incidence of TLF. Subgroup analysis of the two-stent strategy subgroup showed a lower rate of TLF with the use of final kissing balloon inflation (Figure S2). This decrease in the rate of TLF was statistically significant for the two-stent subgroup of the LAD bifurcation group, but not for the two-stent subgroup of the non-LAD bifurcation group.

Another explanation of the high risk of TLF in our study is less frequent use of IVUS-guided PCI for the two-stent strategy in the non-LAD bifurcation group compared with the LAD bifurcation group (49.4% vs. 37.9%, respectively). IVUS-guided PCI is an evidence-based technique used for treating complex lesions [27]. An unprotected LM disease [28] is one such complex lesion that has been shown in a meta-analysis to benefit from IVUS-guided PCI in terms of reduced early major adverse cardiovascular events and late cardiac death [29]. In contrast to previous studies, we did not find improved outcomes with the use of IVUS (Figure S3) in subgroups treated with the two-stent strategy, but it can be postulated that the outcomes may improve because of more optimized intervention in patients receiving IVUS-guided PCI. Our subgroup analysis showed decreased post-PCI residual stenosis in the MB ostium (3.1 ± 5.9% vs. 4.7 ± 7.7%, *p* = 0.018) and SB ostium (10.2 ± 11.9% vs. 7.3 ± 12.5%, *p* = 0.015) in the IVUS group using the two-stent strategy (Table S2). There was also a significant decrease in post-PCI residual stenosis in the parent vessel (PV) (11.3 ± 10.8% vs. 9.6 ± 9.5%, *p* < 0.01) and MB (6.2 ± 10.1% vs. 4.4 ± 8.6%, *p* < 0.01) in bifurcation lesions treated with the IVUS-guided one-stent strategy.

The third possible reason for the high risk of TLF in our study is the difference in angles between the PV, MB, and SB in different locations. The angle between the PV and MB was wider in the non-LAD bifurcation group compared to the LAD bifurcation group (151.8 ± 17.3 vs. 147.4 ± 17.0, respectively, *p* < 0.01) (Table S3). Similarly, the angle between the MV and SB was wider in the non-LAD bifurcation group compared to the LAD bifurcation group (60.9 ± 21.0 vs. 55.0 ± 18.2, respectively, *p* < 0.01) (Table S3). Collins et al. [30] reported a lower rate of major adverse cardiovascular events and chest pain in bifurcation lesions with a narrower angle (<50 degrees) treated with the culotte or crush technique. We did not find an association between the bifurcation angle and long-term outcome in the one-stent group. In contrast, Ki et al. [31] found an angle wider than 152 degrees between the PV and MB to predict TLF in LM bifurcation lesions treated with the crush technique, but not those treated with the T-stenting technique. Using this cut-off of 153 degrees, a similar relationship was observed in our registry in the non-LAD bifurcation group treated with the two-stent strategy, but not in the LAD bifurcation group treated with the same strategy (Figure S4).

Based on the results, our analysis suggests that there is a possible impact of the locations of bifurcations lesions on outcomes of PCI with different stent strategies. Some factors as possible explanations for the difference mentioned above are technical or anatomical. Further future study(s) with better design of prospective manner and considerations for such factors might be necessary.

### Study Limitations

This was a retrospective study based on registry data. Due to the retrospective study design, selection bias cannot be ruled out. We identified many confounding factors and attempted to control for them using propensity score matching. However, these findings should be confirmed in a prospective, randomized, controlled study design to eliminate the possibility of unknown confounding factors.

The dichotomous classification into one-stent and two-stent strategies may be unrealistic in clinical practice. Due to the complex and variable anatomy, many variations of interventions are used to treat bifurcation lesions. In multicenter registries such as COBIS II, many steps of the procedures are modified at the discretion of the operators. This variation may be minimized by using a uniform study design in future studies.

In addition, the sample size of the non-LAD group, which was treated with elective two-stenting strategies (n = 66), is relatively small. Although there was significant statistical difference in the outcomes according to the different stenting strategies, it is necessary to explore whether such difference is consistently observed in other studies with sufficient sample size for the comparison.

Since the COBIS II registry was active between 2003 and 2009, the interventional procedures used in the registry have improved since the collection of the data. Some features of PCI techniques in the COBIS II registry are not contemporary. These include, for example, routine final kissing balloon, proximal optimization technique, or newer stenting techniques such as double kissing crush. In addition, there was an improvement in the quantitative coronary angiography (QCA) technique. QCA provides accurate anatomical details of complex bifurcation lesions and allows better pre-procedure analysis of the lesion. QCA has evolved since the establishment of our registry and now includes dedicated analysis algorithms and three-dimensional analysis of bifurcations [32]. Although the results of QCA often do not match those of other imaging modalities [33,34], QCA is currently widely used for assessing complex anatomy in bifurcation lesions. Use of the aforementioned techniques may have improved the interventional procedures and analysis of bifurcations after the collection of this study’s data.

Stent technology has also improved since the collection of data for this registry. Most procedures in the registry used first-generation DESs. Newer, second-generation DESs have better physical and pharmacological profiles, so these study results will need to be verified using second-generation DESs.

## 5. Conclusions

The outcomes for non-LM bifurcation lesions varied with the location of bifurcation and the treatment strategies used. In non-LAD bifurcation lesions, the outcomes were worse in groups treated with the elective two-stent strategy. In LAD bifurcation lesions, however, the outcomes were similar between groups treated with the planned one-stent and the elective two-stent strategies.

## Figures and Tables

**Table 1:** Baseline and procedural characteristics.

	LAD Bifurcation (n = 1551)			Non-LAD Bifurcation (n = 4930)		
	Planned 1 (n = 1191)	Elective 2 (n = 360)	*p* Value	Planned 1 (n = 427)	Elective 2 (n = 66)	*p* Value
Age, years	63.0 (55.0;69.0)	62.0 (53.0;69.0)	0.2	62.0 (54.0;69.0)	62.0 (53.0;70.0)	0.842
>65	477 (40.1%)	143 (39.7%)	0.96	160 (37.5%)	27 (40.9%)	0.69
Male	841 (70.6%)	252 (70.0%)	0.875	311 (72.8%)	44 (66.7%)	0.373
Acute coronary syndrome	780 (65.5%)	230 (63.9%)	0.62	286 (67.0%)	43 (65.2%)	0.878
Diabetes mellitus	325 (27.3%)	96 (26.7%)	0.869	120 (28.1%)	26 (39.4%)	0.085
Hypertension	671 (56.3%)	201 (55.8%)	0.913	265 (62.1%)	41 (62.1%)	1
Dyslipidemia	371 (31.2%)	103 (28.6%)	0.395	164 (38.4%)	20 (30.3%)	0.258
Smoking	325 (27.3%)	87 (24.2%)	0.268	112 (26.2%)	13 (19.7%)	0.325
Family history of CAD	28 (2.4%)	7 (1.9%)	0.801	18 (4.2%)	3 (4.5%)	1
Peripheral vascular disease	12 (1.0%)	1 (0.3%)	0.317	6 (1.4%)	1 (1.5%)	1
Previous MI	53 (4.5%)	23 (6.4%)	0.176	33 (7.7%)	7 (10.6%)	0.579
Previous CABG	2 (0.2%)	2 (0.6%)	0.498	9 (2.1%)	1 (1.5%)	1
Previous PCI	113 (9.5%)	52 (14.4%)	0.01	57 (13.3%)	15 (22.7%)	0.069
Previous Cerebrovascular event	63 (5.3%)	19 (5.3%)	1	20 (4.7%)	5 (7.6%)	0.487
Chronic kidney disease	33 (2.8%)	8 (2.2%)	0.703	7 (1.6%)	2 (3.0%)	0.771
LVEF	60.0 (51.0;65.0)	61.0 (54.0;66.7)	<0.01	58.5 (53.0;63.0)	60.0 (54.8;68.0)	0.254
EF < 50	223 (21.9%)	53 (18.2%)	0.198	60 (17.2%)	9 (17.0%)	1
Multivessel disease	493 (41.4%)	156 (43.3%)	0.553	236 (55.3%)	44 (66.7%)	0.108
Medina classification			<0.01			<0.01
True bifurcation	633 (53.1%)	274 (76.1%)		181 (42.4%)	56 (84.8%)	
−1,1,1	385 (32.3%)	159 (44.2%)		117 (27.4%)	30 (45.5%)	
−1,0,1	93 (7.8%)	24 (6.7%)		37 (8.7%)	6 (9.1%)	
−0,1,1	155 (13.0%)	91 (25.3%)		27 (6.3%)	20 (30.3%)	
Nontrue bifurcation	558 (46.9%)	86 (23.9%)		246 (57.6%)	10 (15.2%)	
−0,0,1	13 (1.1%)	36 (10.0%)		8 (1.9%)	3 (4.5%)	
−0,1,0	210 (17.6%)	23 (6.4%)		80 (18.7%)	2 (3.0%)	
−1,0,0	157 (13.2%)	7 (1.9%)		93 (21.8%)	1 (1.5%)	
−1,1,0	178 (14.9%)	20 (5.6%)		65 (15.2%)	4 (6.1%)	
MB or SB calcification	244 (20.5%)	76 (21.1%)	0.855	36 (8.4%)	4 (6.1%)	0.679
Main branch total occlusion	146 (12.3%)	23 (6.4%)	<0.01	61 (14.3%)	10 (15.2%)	1
Side branch total occlusion	44 (3.7%)	20 (5.6%)	0.16	34 (8.0%)	8 (12.1%)	0.374
Stent type			0.014			0.538
- SES	570 (47.9%)	206 (57.2%)		174 (40.7%)	31 (47.0%)	
- PES	340 (28.5%)	90 (25.0%)		139 (32.6%)	20 (30.3%)	
- EES	123 (10.3%)	27 (7.5%)		52 (12.2%)	10 (15.2%)	
- ZES	136 (11.4%)	28 (7.8%)		53 (12.4%)	4 (6.1%)	
- Others	22 (1.8%)	9 (2.5%)		9 (2.1%)	1 (1.5%)	
Stenting technique			<0.01			<0.01
One stent	1190 (100.0%)	0 (0.0%)		427 (100.0%)	0 (0.0%)	
Crush	0 (0.0%)	40 (11.2%)		0 (0.0%)	7 (10.6%)	
Culottes	0 (0.0%)	6 (1.7%)		0 (0.0%)	3 (4.5%)	
Kissing	0 (0.0%)	32 (8.9%)		0 (0.0%)	4 (6.1%)	
Mini crush	0 (0.0%)	154 (43.0%)		0 (0.0%)	25 (37.9%)	
T-stent	0 (0.0%)	126 (35.2%)		0 (0.0%)	27 (40.9%)	
Final kissing balloon inflation	390 (32.7%)	296 (82.2%)	<0.01	112 (26.2%)	52 (78.8%)	<0.01
Guidance of intravascular ultrasound	347 (29.1%)	178 (49.4%)	<0.01	87 (20.4%)	25 (37.9%)	<0.01
Transradial intervention	282 (23.7%)	68 (18.9%)	0.067	119 (27.9%)	11 (16.7%)	0.076
Main branch						
MB Total stent length, mm	28.0 (23.0;33.0)	28.0 (23.0;33.0)	0.011	24.0 (20.0;32.0)	28.0 (23.0;33.0)	0.214
Maximal stent diameter, mm	3.0 (3.0; 3.5)	3.0 (3.0; 3.5)	0.135	3.0 (2.8; 3.0)	3.0 (2.8; 3.0)	0.574
SB stenting	34 (2.9%)	360 (100%)	<0.01	29 (6.8%)	66 (100%)	<0.01
Side branch						
SB total stent length *, mm	20.0 (16.0;28.0)	20.0 (16.0;28.0)	0.946	24.0 (18.0;32.0)	20.0 (18.0;28.0)	0.316
Maximal stent diameter, mm	2.8 (2.5; 2.8)	2.8 (2.5;3.0)	0.432	3.0 (2.8; 3.0)	2.8 (2.5; 3.0)	<0.01

Values are mean ± SD or median (25 percentile, 75 percentiles) according to distribution. CABG = coronary artery bypass grafting; CAD = coronary artery disease; EES = everolimus-eluting stent(s); LAD = left anterior descending artery; LVEF = left ventricle ejection fraction; MB = main branch; MI = myocardial infarction; PCI = percutaneous coronary intervention; PES = paclitaxel-eluting stent(s); SB = side branch; SES = sirolimus-eluting stent(s); ZES = zotarolimus-eluting stent(s). * Only lesions with side branch stenting were compared.

**Table 2:** Quantitative coronary angiographic analysis.

	LAD Bifurcation (n = 1551)			Non-LAD Bifurcation (n = 493)		
	Planned 1 (n = 1191)	Elective 2 (n = 360)	*p* Value	Planned 1 (n = 427)	Elective 2 (n = 66)	*p* Value
Pre-intervention						
PV-MB angle	147.6 ± 16.7	146.9 ± 17.9	0.505	151.4 ± 17.6	154.5 ± 15.1	0.176
PV-SB angle	152.0 ± 18.8	155.2 ± 16.9	<0.01	142.0 ± 21.3	146.0 ± 18.5	0.151
MB-SB angle	55.8± 18.6	52.1 ± 16.6	<0.01	61.6 ± 21.0	56.5 ± 20.4	0.067
PV RD, mm	3.3 ± 0.5	3.3 ± 0.5	0.952	3.2 ± 0.5	3.3 ± 0.5	0.36
MB RD, mm	2.7 ± 0.4	2.6 ± 0.4	<0.01	2.6 ± 0.4	2.6 ± 0.5	0.345
SB RD, mm	2.4 ± 0.3	2.4 ± 0.3	0.012	2.5 ± 0.3	2.4 ± 0.3	0.05
PV MLD, mm	1.6 ± 0.8	1.7 ± 0.8	<0.01	1.4 ± 0.9	1.7 ± 0.8	<0.01
MB Ostium MLD, mm	1.3 ± 0.7	1.3 ± 0.7	0.967	1.4 ± 0.7	1.1 ± 0.6	<0.01
MB Ostium Diameter Stenosis (%)	52.4 ± 23.2	50.7 ± 23.5	0.223	47.8 ± 24.3	56.8 ± 24.1	<0.01
SB Ostium MLD, mm	1.4 ± 0.6	1.0 ± 0.5	<0.01	1.5 ± 0.6	0.9 ± 0.6	<0.01
SB Ostium Diameter Stenosis (%)	43.8 ± 22.3	58.4 ± 20.3	<0.01	38.0 ± 23.7	60.7 ± 23.9	<0.01
MB lesion length, mm	19.1 ± 11.5	20.2± 13.2	0.159	17.4 ± 10.5	21.7 ± 12.1	<0.01
SB lesion length, mm	4.4 ± 6.2	10.8± 8.5	<0.01	2.9 ± 5.1	12.3 ± 10.7	<0.01
Post-intervention						
PV Residual Stenosis (%)	10.3 ± 9.8	6.9 ± 9.1	<0.01	12.4 ± 12.0	10.8 ± 9.0	0.199
MB Ostium Residual Stenosis (%)	4.7 ± 7.6	3.9 ± 7.0	0.079	8.6 ± 13.7	4.4 ± 6.6	<0.01
MB Distal Residual Stenosis (%)	7.7 ± 18.0	4.3 ± 10.9	<0.01	9.5 ± 19.0	8.5 ± 20.9	0.67
SB Ostium Residual Stenosis (%)	43.7 ± 21.5	8.9 ± 12.5	<0.01	37.0 ± 22.2	8.3 ± 10.9	<0.01
SB distal Residual Stenosis (%)	25.5 ± 19.5	5.3 ± 7.6	<0.01	20.4 ± 19.3	4.6 ± 8.5	<0.01

Values were expressed as the mean ± SD. MLD = minimal lumen diameter, MB = main branch; PV = proximal vessel, RD = reference diameter, SB = side branch.

**Table 3:** Outcomes and hazard ratio for clinical outcomes according to location of bifurcation lesions and stenting strategies.

	LAD Bifurcation (n = 1551)							Non-LAD Bifurcation (n = 493)						
	Planned 1 (n = 1191) n (%)	Elective 2 (n = 360) n (%)	*p* Value	Adjusted HR (95% CI)	*p* Value	PS Matching Adjusted HR (95% CI)	*p* Value	Planned 1 (n = 427) n (%)	Elective 2 (n = 66) n (%)	*p* Value	Adjusted HR (95% CI)	*p* Value	PS Matching Adjusted HR (95% CI)	*p* Value
Target lesion failure	103 (8.6%)	37 (10.3%)	0.40	1.37 (0.88–2.12)	0.15	0.96 (0.62–1.47)	0.85	27 (6.3%)	15 (22.7%)	<0.01	4.34 (1.93–9.76)	<0.01	2.51 (1.04–6.09)	0.04
Cardiac death	11 (0.9%)	3 (0.8%)	1	1.14 (0.27–4.83)	0.84	0.91 (0.23–3.61)	0.9	5 (1.2%)	1 (1.5%)	1	1.59 (0.18–13.6)	0.62	0.002 (2.67 × 10^−191^–3.03 × 10^185^)	0.97
AMI	15 (1.3%)	6 (1.7%)	0.74	2.00 (0.65–6.14)	0.22	0.82 (0.27–2.47)	0.73	7 (1.6%)	5 (7.6%)	0.013	5.43(0.95–31.03)	0.057	3.54 (0.81–15.4)	0.09
Target lesion revascularization	84 (7.1%)	32 (8.9%)	0.29	1.47 (0.91–2.36)	0.1	1.04 (0.65–1.67)	0.84	20 (4.7%)	11 (16.7%)	<0.01	4.30(1.64–11.27)	<0.01	3.39 (1.01–11.28)	0.04
Target vessel revascularization	123 (10.3%)	41 (11.4%)	0.63	1.21 (0.77–1.76)	0.44	0.96 (0.64–1.45)	0.88	27 (6.3%)	12 (18.2%)	<0.01	5.07 (1.69–9.74)	<0.01	3.41 (1.11–10.44)	0.03

AMI = Acute myocardial infarction; CI = Confidence interval; PS = propensity score; HR = Hazard ratio.

## Data Availability

Not applicable.

## Supplementary Materials

The following supporting information can be downloaded at: https://www.mdpi.com/article/10.3390/jcm11195658/s1, Figure S1: Standardized mean difference before and after matching was shown for (a) LAD bifurcation lesion group, (b) non-LAD bifurcation lesion group. Most variables are matched with SMD lower than 0.1. Figure S2: Kaplan-Meier curves for TLF according to final kissing balloon with elective two-stent strategy in (a) LAD and non-LAD bifurcation lesions, (b) LAD bifurcation and c) non-LAD bifurcation lesions. Figure S3: Kaplan-Meier curves for TLF according to IVUS guidance with elective two-stent strategy in (a) LAD and non-LAD bifurcation lesions, (b) LAD bifurcation and (c) non-LAD bifurcation lesions. Figure S4: Kaplan-Meier curves for TLF according to PV-MB angle in bifurcation lesions treated with elective two-stent strategy in (a) LAD bifurcation lesions and (b) non-LAD bifurcation lesions. Table S1. Mean difference, standard deviation and SMD after propensity score matching. Table S2. QCA according to treatment strategies and IVUS usage. Table S3. QCA according to lesion location.
