# Supplementary material for: Effect of Stenting Strategy on the Outcome in Patients with Non-Left Main Bifurcation Lesions
Source: J Clin Med. 2022 Sep 26;11(19):5658. doi: 10.3390/jcm11195658 (PMC9571815; doi:10.3390/jcm11195658)
Supplement: Supplementary file 1 [file jcm-11-05658-s001.zip › jcm-1901087-supplementary.pdf]

Supplementary Material

APPENDIX

CONTENTS

Supplementary Methods

Supplementary Tables

Supplementary Figures and Figure Legends

Supplementary Methods

Percutaneous coronary intervention

Patients who had percutaneous coronary intervention (PCI) were pre-treated with loading doses of aspirin (300 mg) and clopidogrel (300-600 mg) unless these antiplatelet medications had previously been administered. Low molecular weight heparin or unfractionated heparin was used during PCI to achieve an activated clotting time of 250–300 seconds. Access, type of drug-eluting stents, use of intravascular ultrasound or glycoprotein IIb/IIIa receptor inhibitors, stenting techniques for bifurcation lesions were at the operator's discretion. The duration of the dual antiplatelet medications were at least six months, and aspirin was prescribed indefinitely.

Supplementary Figures and Figure Legends

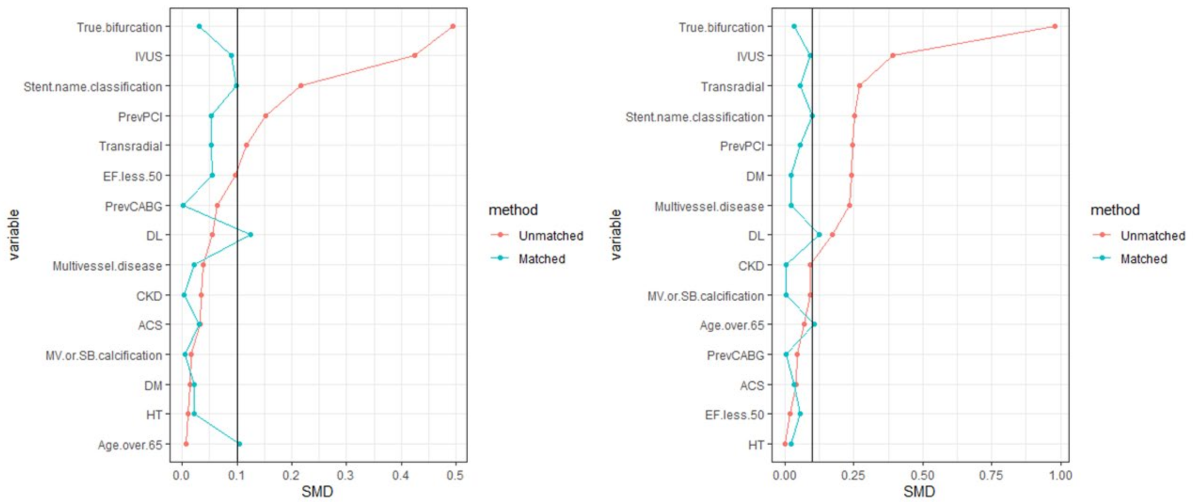

(a) (b)

Figure S1, Standardized mean difference before and after matching was shown for a) LAD bifurcation lesion group, b) non-LAD bifurcation lesion group. Most variables are matched with SMD lower than 0.1.

LAD= left anterior descending; IVUS= intravascular ultrasound; PCI= percutaneous coronary intervention; EF= ejection fraction; PrevCABG= previous coronary artery bypass graft surgery; DL= dyslipidemia; CKD= chronic kidney disease; ACS= acute coronary syndrome; DM= diabetes mellitus; HT= hypertension

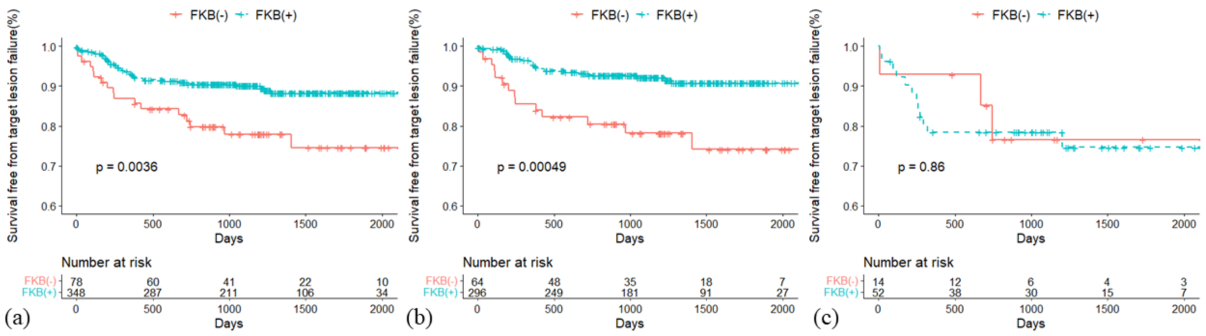

Figure S2, Kaplan-Meier curves for TLF according to final kissing balloon with elective two-stent strategy in a) LAD and non-LAD bifurcation lesions, b) LAD bifurcation and c) non-LAD bifurcation lesions

LAD= left anterior descending; FKB= final kissing balloon; TLF= target lesion failure

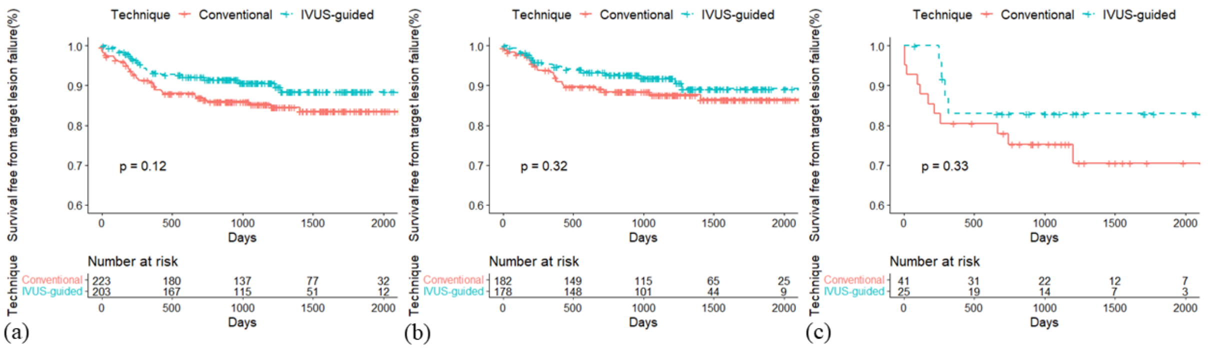

Figure S3, Kaplan-Meier curves for TLF according to IVUS guidance with elective two-stent strategy in a) LAD and non-LAD bifurcation lesions, b) LAD bifurcation and c) non-LAD bifurcation lesions

IVUS= intravascular ultrasound; LAD= left anterior descending; TLF= target lesion failure

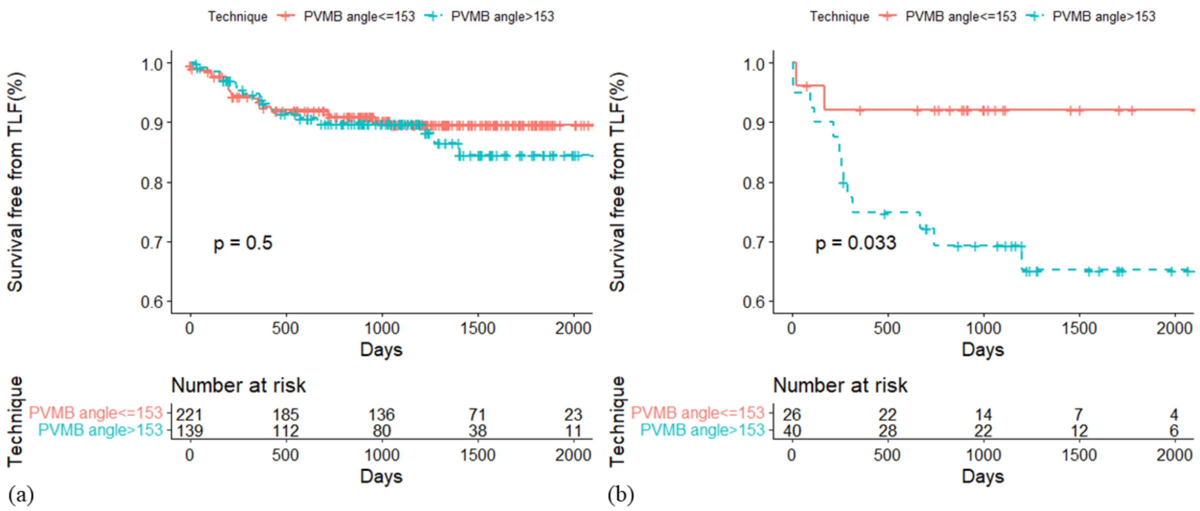

Figure S4, Kaplan-Meier curves for TLF according to PV-MB angle in bifurcation lesions treated with elective two-stent strategy in a) LAD bifurcation lesions and b) non-LAD bifurcation lesions

LAD= left anterior descending; MB= main branch; PV= parent vessel; TLF= target lesion failure

Supplementary Tables

Table S1. Mean difference, standard deviation and SMD after propensity score matching

	**LAD Bifurcation (n = 1,551)**			**Non-LAD Bifurcation (n = 493)**		
	**Planned 1 (n=613)**	**Elective 2 (n=340)**	**SMD**	**Planned 1 (n=112)**	**Elective 2 (n=57)**	**SMD**
Age>65 (mean (SD))	0.41 (0.49)	0.41 (0.49)	0.005	0.46 (0.50)	0.40 (0.49)	0.104
Acute coronary syndrome (mean (SD))	0.64 (0.48)	0.63 (0.48)	0.028	0.63 (0.48)	0.65 (0.48)	0.031
Diabetes mellitus (mean (SD))	0.26 (0.44)	0.27 (0.44)	0.022	0.38 (0.49)	0.39 (0.49)	0.022
Hypertension (mean (SD))	0.56 (0.50)	0.56 (0.50)	0.011	0.61 (0.49)	0.60 (0.49)	0.022
Dyslipidemia (mean (SD))	0.31 (0.46)	0.30 (0.46)	0.029	0.26 (0.44)	0.32 (0.47)	0.125
Chronic kidney disease (mean (SD))	0.02 (0.15)	0.02 (0.15)	0.006	0.04 (0.19)	0.04 (0.19)	0.003

Previous CABG (mean (SD))	0.00 (0.06)	0.00 (0.05)	0.006	0.02 (0.13)	0.02 (0.13)	0.002
Previous PCI (mean (SD))	0.13 (0.34)	0.13 (0.34)	0.008	0.20 (0.40)	0.18 (0.38)	0.054
LVEF<50% (mean (SD))	0.16 (0.36)	0.16 (0.37)	0.022	0.18 (0.38)	0.16 (0.37)	0.055
Stent type (%)			0.051			0.099
EES	44(7.2)	27 (7.9)		14 (12.5)	8 (14.0)	
PES	157 (25.6)	90 (26.5)		38 (33.9)	19 (33.3)	
SES	343 (56.0)	189 (55.6)		52 (46.4)	25 (43.9)	
ZES	55 (9.0)	27 (7.9)		7 (6.2)	4 (7.0)	
Others	14 (2.3)	7 (2.1)		1 (0.9)	1 (1.8)	
Multivessel disease (mean (SD))	0.42 (0.49)	0.42 (0.49)	0.006	0.62 (0.49)	0.61 (0.49)	0.022
Main branch or side branch calcification (mean (SD))	0.22 (0.41)	0.20 (0.40)	0.038	0.07 (0.26)	0.07 (0.26)	0.005

True bifurcation (mean (SD))	0.71 (0.45)	0.75 (0.44)	0.073	0.81 (0.39)	0.82 (0.38)	0.031
Guidance of intravascular ultrasound (mean (SD))	0.39 (0.49)	0.46 (0.50)	0.152	0.24 (0.43)	0.28 (0.45)	0.09
Transradial (mean (SD))	0.16 (0.37)	0.20 (0.40)	0.084	0.20 (0.40)	0.18 (0.38)	0.054

Values were expressed as the mean difference (SD)

CAD= coronary artery disease; EES= everolimus-eluting stent(s); LAD = left anterior descending artery; LVEF= left ventricle ejection fraction; MI= myocardial infarction; PCI= percutaneous coronary intervention; PES = paclitaxel-eluting stent(s); SB= side branch; SD= standard deviation; SES = sirolimus-eluting stent(s); ZES = zotarolimus-eluting stent(s)

Table S2. QCA according to treatment strategies and IVUS usage

	Planned 1			Elective 2		
	**Conventional (n=1184)**	**IVUS-guided (n=434)**	**p**	**Conventional (n=223)**	**IVUS-guided (n=203)**	**p**
Pre-intervention						
PV-MB angle	148.2± 17.5	149.4±15.7	0.19	149.4± 17.4	146.5±17.9	0.09
PV-SB angle	149.4± 19.5	149.5±21.2	0.92	154.0± 18.2	153.4±16.7	0.72
PV RD, mm	57.3 ± 19.3	57.6 ± 19.7	0.77	50.7 ± 16.2	55.1 ± 18.1	<0.01
MB RD, mm	3.3 ± 0.5	3.2 ± 0.5	0.41	3.3 ± 0.5	3.2 ± 0.5	0.12
SB RD, mm	2.7 ± 0.4	2.7 ± 0.5	0.53	2.6 ± 0.5	2.6 ± 0.4	0.80
PV MLD, mm	2.4 ± 0.3	2.4 ± 0.3	0.63	2.4 ± 0.3	2.4 ± 0.3	0.76
MB Ostium MLD, mm	1.5 ± 0.9	1.6 ± 0.8	0.015	1.8 ± 0.8	1.7 ± 0.8	0.21
MB Ostium Diameter Stenosis (%)	1.3 ± 0.7	1.3 ± 0.6	0.29	1.2 ± 0.7	1.3 ± 0.7	0.57
SB Ostium MLD, mm	51.4 ± 24.2	50.1 ± 23.2	0.32	51.9 ± 24.7	50.4 ± 25.1	0.54
SB Ostium Diameter Stenosis (%)	1.4 ± 0.6	1.5 ± 0.6	0.015	1.0 ± 0.5	1.0 ± 0.5	0.57
MB lesion length, mm	43.0 ± 23.5	39.9 ± 21.4	0.012	59.3 ± 21.8	58.1 ± 20.1	0.56

SB lesion length, mm	18.6 ± 11.4	18.8 ± 11.0	0.78	21.2 ± 12.5	19.5 ± 13.6	0.17
PV RD, mm	4.2 ± 6.0	3.6 ± 5.8	0.06	10.9 ± 8.8	11.3 ± 9.0	0.62
Post-intervention						
PV Residual Stenosis (%)	11.3 ± 10.8	9.6 ± 9.5	<0.01	8.3 ± 9.3	6.7 ± 9.0	0.094
MB Ostium Residual Stenosis (%)	6.2 ± 10.1	4.4 ± 8.6	<0.01	4.7 ± 7.7	3.1 ± 5.9	0.018
MB Distal Residual Stenosis (%)	8.6 ± 18.5	7.2 ± 17.5	0.18	4.9 ± 11.2	5.1 ± 14.8	0.87
SB Ostium Residual Stenosis (%)	42.1 ± 22.1	41.4 ± 21.4	0.55	10.2 ± 11.9	7.3 ± 12.5	0.015
SB distal Residual Stenosis (%)	24.2 ± 19.8	23.8 ± 18.9	0.71	5.8 ± 7.7	4.6 ± 7.8	0.11

Values were expressed as the mean ± SD.

MB= main branch; MLD = minimal lumen diameter; PV= proximal vessel; RD = reference diameter; SB = side branch

Table S3. QCA according to lesion location

	LAD Bifurcation (n = 1,551)	Non-LAD Bifurcation (n = 493)	P value
Pre-intervention			
PV-MB angle	147.4 ± 17.0	151.8 ± 17.3	<0.01
PV-SB angle	152.8 ± 18.4	142.6 ± 21.0	<0.01
MB-SB angle	55.0 ± 18.2	60.9 ± 21.0	<0.01
PV RD, mm	3.3 ± 0.5	3.2 ± 0.5	0.673
MB RD, mm	2.7 ± 0.4	2.6 ± 0.4	0.379
SB RD, mm	2.4 ± 0.3	2.5 ± 0.3	<0.01
PV MLD, mm	1.6 ± 0.8	1.4 ± 0.9	<0.01
MB Ostium MLD, mm	1.3 ± 0.7	1.4 ± 0.7	0.057
MB Ostium Diameter Stenosis (%)	51.8 ± 23.9	48.8 ± 24.7	0.018
SB Ostium MLD, mm	1.3 ± 0.6	1.5 ± 0.7	<0.01
SB Ostium Diameter Stenosis (%)	47.1 ± 22.9	40.9 ± 25.1	<0.01
MB lesion length, mm	19.3 ± 11.9	18.0 ± 10.8	0.016

SB lesion length, mm	5.9 ± 7.3	4.2 ± 6.9	0
Post-intervention			
PV Residual Stenosis (%)	9.5 ± 9.7	12.2 ± 11.6	<0.01
MB Ostium Residual Stenosis (%)	4.5 ± 7.5	8.0 ± 13.0	<0.01
MB Distal Residual Stenosis (%)	6.9 ± 16.6	9.4 ± 19.2	0.011
SB Ostium Residual Stenosis (%)	35.5 ± 24.7	33.1 ± 23.2	0.056
SB distal Residual Stenosis (%)	20.8 ± 19.4	18.2 ± 19.0	0.012

Values were expressed as the mean ± SD.

MB= main branch; MLD = minimal lumen diameter; PV= proximal vessel; RD = reference diameter; SB = side branch
